# Nematode–Microbe Complexes in Soils Replanted with Apple

**DOI:** 10.3390/microorganisms10010157

**Published:** 2022-01-12

**Authors:** Xorla Kanfra, Andreas Wrede, Julia Moll, Holger Heuer

**Affiliations:** 1 Julius Kühn Institute (JKI)—Federal Research Centre for Cultivated Plants, Institute for Epidemiology and Pathogen Diagnostics, 38104 Braunschweig, Germany; xorla.kanfra@julius-kuehn.de; 2 Department of Horticulture, Landwirtschaftskammer Schleswig-Holstein, 25373 Ellerhoop, Germany; awrede@lksh.de; 3 Helmholtz Centre for Environmental Research—UFZ, Department of Soil Ecology, 06120 Halle (Saale), Germany; julia.moll@ufz.de

**Keywords:** replant disease, malus, free-living nematodes, bacteria, fungi, rhizosphere, nematode–microbe association, disease complex, metabarcoding, nematode community

## Abstract

Apple replant disease is a severe problem in orchards and tree nurseries. Evidence for the involvement of a nematode–microbe disease complex was reported. To search for this complex, plots with a history of apple replanting, and control plots cultivated for the first time with apple were sampled in two fields in two years. Shoot weight drastically decreased with each replanting. Amplicon sequencing of the nematode community and co-extracted fungal and bacterial communities revealed significant differences between replanted and control plots. Free-living nematodes of the genera *Aphelenchus* and *Cephalenchus* and an unidentified Dorylaimida were associated with replanted plots, as indicated by linear discriminant analysis effect size. Among the co-extracted fungi and bacteria, *Mortierella* and *Methylotenera* were most indicative of replanting. Some genera, mostly *Rhabditis*, *Streptomyces* and a fungus belonging to the Chaetomiaceae indicated healthy control plots. Isolating and investigating the putative disease complexes will help to understand and alleviate stress-induced root damage of apple in replanted soil.

## 1. Introduction

Continuous monoculture is commonly associated with intensive agricultural production and may result in negative plant–soil feedbacks leading to yield decline of crops [1]. Replant disease was described as the phenomenon that soil gradually loses its capacity to support the growth of a specific plant after replanting without any known pathogen causing the decline [1,2]. For apple, the phenomenon has been termed specific sickness [3] or specific apple replant disease (ARD) [4] to distinguish it from root damage by the endoparasitic nematode *Pratylenchus penetrans*. Affected plants show significantly reduced shoot growth, root cell necrosis and patchy blackening of root cells, impaired root hair development and low cell vitality which may lead to root death [5]. Fruit yield and quality are significantly reduced [6]. ARD is a worldwide phenomenon affecting various apple-growing regions, yet the causes are still not clear [7,8,9].

Mitigating measures such as soil pasteurization or fumigation significantly improved the growth of apple plants which gives evidence that the disease is caused by biotic factors [10]. Accumulation of phenolic compounds or phytotoxins in disease-affected roots has been discussed to play a role in the disease [11,12]. Recently, a transcriptomic analysis of the molecular responses of apple plants to ARD soils showed peculiar defense reactions to biotic stress, especially up-regulation of genes for phytoalexin synthesis but failed to point to the specific biotic origin of the disease [13]. Depending on the study, various biotic agents including the oomycetes *Pythium* and *Phytophthora,* the fungi *Cylindrocarpon/Ilyonectria*, *Fusarium* and *Rhizoctonia*, or bacteria of the genera *Bacillus*, *Pseudomonas*, and *Streptomyces* have been implicated with ARD-related growth depression [6,14,15,16]. The migratory endoparasitic nematode *P. penetrans* was frequently found in affected orchards, but the abundance was often below damage thresholds or did not correlate with disease symptoms [12,17]. It was observed as a characteristic of ARD that apple trees recover after transfer to healthy soil [1], thus putative ARD causing biota seem to reside in soil and interact with the roots in the rhizosphere. To identify biotic causes outside the root, high throughput sequencing data of bacterial and fungal communities in ARD-affected and healthy soils were compared [18]. Significant differences were revealed, including elevated levels of nematode-associated bacteria and fungi in ARD soil, but particular causative agents have not been identified yet. Extracellular compounds released by fungi in the apple rhizosphere were shown to affect apple roots [15,19]. Thus, biotic interactions in the rhizosphere of apple plants may lead to external production of deleterious compounds that trigger ARD. The involvement of nematodes in such interactions has not been investigated so far. Some species of soil nematodes are involved in interactions with microbes that affect higher organisms such as insects [20], livestock [21] or plants [22,23].

Free-living nematodes in soil include bacterivores, fungivores, predators, omnivores and root feeders [24]. In contrast to endoparasitic nematodes, they are not well studied. Evidence is mounting that nematodes contribute to ARD. Heating to 50 °C [25], planting of *Tagetes* [26,27] or application of nematicides [28,29] were treatments of ARD soils that targeted nematodes and ameliorated the disease. Recently, we showed that the nematode fraction extracted from ARD soil induced root symptoms typical for ARD, like reduced weight, increased levels of phenolic compounds and phytoalexins and browning [30]. We separated nematodes and microbes from ARD soils (replanted) or control soils (first time planted with apple) from plots of the same field and inoculated the fractions in combinations or singly to ARD-susceptible apple rootstocks. The microbial fraction from ARD soil and control soil enhanced the disease symptoms when added to the nematodes, but had only minor effects without the nematodes. The nematode fractions were thoroughly washed with sterile water but contained the microbes that were strongly associated with the nematode bodies. As we did not detect significant numbers of plant-parasitic nematodes in the ARD soils and not more than in the control soils, we concluded that particular free-living nematodes in association with certain soil microbes induced the disease symptoms. When ARD soils were pre-cultivated with Tagetes, the extracted nematode fraction did not induce ARD anymore [27].

The objective of this study was to compare nematode communities and nematode-associated microbial communities extracted from ARD plots and control plots in two fields and two years. The study was done with soils from two experimental fields, where plots were either frequently replanted or planted the first time with apple rootstocks, and known pathogens of apple did not play a significant role [26,30,31,32,33]. We hypothesized that differences in the community structure of free-living nematodes and the nematode-associated fungi or bacteria between ARD and healthy soils will reveal putative disease complexes that are associated with the induction of ARD symptoms. We used high-throughput amplicon sequencing to characterize the nematode and nematode-associated microbial communities.

## 2. Materials and Methods

### 2.1. Field Soil Sample Collection and Nematode Extraction

Soils from ARD reference field sites Heidgraben (53.6992° N 9.6832° E) and Ellerhoop (53.7143° N 9.7701° E), both of which were located in the tree nursery area around Pinneberg, Germany, were used for this study. At Heidgraben, an Entic Podzol (according to WRB 2015) had developed from aeolian sand. The soil in Ellerhoop was classified as an Endostagnic Luvisol from glacial till. On the field plots, the apple rootstock “Bittenfelder Sämling” was repeatedly planted and uprooted at the Heidgraben site (planting of ARD plots in 2009, 2010, 2012, 2014, 2016; uprooting in 2009, 2011, 2013, 2015, 2017) and at the Ellerhoop site (planting of ARD plots in 2009, 2011, 2013, 2015, 2017, 2019; uprooting in 2010, 2012, 2014, 2016, 2018, 2020) in four randomly arranged plots interspersed with four control plots covered with grass [11,32,34]. At each uprooting, the shoot fresh mass was determined for each plot. Before soil sampling was started, the grass was removed from the control plots and apple was planted to get a non-replanted control. In Heidgraben, the soil samplings were done in April 2016 (H1) and in April 2017 (H4). In Ellerhoop, we took soil samples in November 2017 (E2) and in April 2018 (E3). Soil samples of each plot were taken at a depth of 0–30 cm from the apple root zone of three individual plants in a zigzag pattern. These constituted 24 samples from each site, sampled at two different time points equaling 96 soil samples in total. Root fresh mass was recorded for samplings E3 and H4. The soil samples were stored at 4 °C for two weeks before nematode extraction. Nematodes were extracted from 250 mL portions of the soils by centrifugal floatation using MgSO_4_ at 1.18 specific density [35]. Nematodes were collected on a 20-µm sieve, thoroughly washed with sterile water, transferred to 50 mL tubes with sterile water, pelleted by centrifugation at 4000× *g* for 20 min. The pellet was frozen at −20 °C until DNA extraction.

### 2.2. Characterization of the Nematode Diversity

Total DNA from each soil nematode pellet was extracted using the FastPrep FP120 bead beating system for 30 s at high speed for cell lysis of nematodes and associated microbes, and the FastDNA SPIN Kit for Soil (MP Biomedicals, Eschwege, Germany) as described by the manufacturer. To characterize the nematode diversity, the primer SSU18SF01 GATCAGGGTTCGACTCCGGAGA (this study) targeting the 18S rRNA genes of nematodes together with the D2B primer AGTTTCCTCTGGCTTCGTCCTGC [36], which targets the D2/D3 expansion segment of the 28S rRNA gene, were used to amplify about 4 kb of the rRNA cistron in a PCR reaction mix containing 10 µL of 5 × Q5 reaction buffer (New England Biolabs, Frankfurt, Germany), 7.5 µL of 2 mM dNTP, 2.5 µL of 2 mg/mL BSA, 10 µL of Q5 High GC Enhancer (New England Biolabs), 0.5 µL of 2 U/mL Q5 High-Fidelity DNA Polymerase (New England Biolabs) and 2.5 µL of each primer (10 µM). Template DNA and water were added to give a final volume of 50 μL for each sample. The PCR was carried out using the following cycling conditions: The thermocycler block was heated for 1 min followed by an initial denaturation of 5 min at 95 °C, 20 cycles of (95 °C for 15 s; 54 °C for 30 s; 68 °C for 2 min) and a final extension at 68 °C for 4 min. In a nested PCR, around 362 bp of 18S rDNA was amplified from the first PCR products using the forward primer NF1 CGTATCGCCTCCCTCGCGCCATCAG and the reverse primer 18Sr2b CTATGCGCCTTGCCAGCCCGCTCAG [37], with Illumina 5′-overhang TCGTCGGCAGCGTCAGATGTGTATAAGAGACAG or GTCTCGTGGGCTCGGAGATGTGTATAAGAGACAG, respectively. PCR reactions of 50 μL contained 10 μL of 5x GoTaq Flexi buffer (Promega, Mannheim, Germany), 7.5 μL 25 mM MgCl_2_, 5 μL of 2 mM dNTP each, 1 μL of each 10 µM primer solution, 2.5 μL of 2 mg/mL BSA, 4 μL of 50% acetamide, 0.4 μL of 5 U/µL GoTaq DNA polymerase (Promega, Mannheim, Germany). The following PCR cycler condition was used: Initial denaturation of 5 min at 94 °C, 25 cycles of (94 °C for 45 s; 54 °C for 30 s; 72 °C for 1 min) and a final extension of 10 min at 72 °C. The resulting PCR product was purified using the High Pure PCR Purification kit (Roche Diagnostics, Rotkreuz, Switzerland) following the manufacturer’s instructions. Barcoded amplicon sequencing of the 18S rRNA genes was done by 2 × 300 bp paired-end high-throughput sequencing (MiSeq Reagent kit v3) on an Illumina MiSeq platform (Illumina, San Diego, CA, USA) at the Department of Soil Ecology of the Helmholtz Centre for Environmental Research—UFZ, Halle, Germany [38].

### 2.3. Characterization of the Nematode-Associated Microbial Diversity

The fungal community associated with the extracted nematodes was amplified using the primers gITS7 and ITS4 targeting the ITS2 region [39]. PCR was performed in a reaction mixture of 50 μL consisting of 10 μL of 5× GoTaq Flexi buffer (Promega, Mannheim, Germany), 5 μL of 25 mM MgCl_2_, 5 μL of 2 mM dNTP, 2.5 μL of 2 mg/mL BSA, 1 μL of each primer (10 μM), 1 μL of 5 U/μL GoTaq polymerase (Promega) and 1 μL of nematode community DNA. The following PCR cycler condition was used: Initial denaturing of 5 min at 94 °C, followed by 30 cycles of (94 °C for 30 s, 56 °C for 30 s, 72 °C for 1 min) and final elongation at 72 °C for 5 min. To characterize the bacterial diversity associated with the extracted nematodes, the V3-V4 regions of 16S rRNA genes were amplified using the primers 341F [40] and 806R [41] in a 25 μL reaction volume containing 2.5 μL of 10× reaction buffer (New England Biolabs, Frankfurt, Germany), 0.125 μL of 5 U/μL NEB HotStart Taq polymerase, 2.5 μL of 2 mM dNTP, 1 μL of 2.5 mM MgCl_2_, 2.5 μL of 2 mg/mL BSA, 1 μL of each primer (10 μM) and 1 μL of nematode community DNA. The following temperature steps were applied: 2 min at 94 °C, 30 cycles of 20 s at 94 °C, 20 s at 56 °C, 40 s at 72 °C, followed by a final elongation for 5 min at 72 °C. Amplicon sequencing of the ITS2 or 16S rRNA genes was done by 2 × 250 bp paired-end high-throughput sequencing on an Illumina HiSeq 2500 platform by Novogene (Cambridge, UK).

### 2.4. Sequence Analysis

The 18S rRNA, ITS or16S rRNA sequence demultiplexing was done using the MiSeq Controller Software and diversity spacers were trimmed using Biopieces (www.biopieces.org, accessed on 1 July 2021). The sequence reads for nematodes and nematode-associated bacteria were processed using USEARCH (v11.0.667). Raw reads were processed using the protocol established in the USEARCH pipeline followed by OTU (operational taxonomic units) clustering using UPARSE [42]. The nematode sequence read preparation and processing included paired-end merging with an overlapping minimum read length of 10 base pairs, filtering of low-quality sequences and dereplication to find unique sequences following default settings. OTU clustering and chimera removal was performed at a 97% identity threshold via the *cluster_otu* command implemented in the UPARSE algorithm. BlastN assigned taxonomic affiliations for the nematodes were done against the Silva SSU 138 database [43]. Processing of the bacteria sequence reads included paired-end merging with an overlapping minimum read length of 10 base pairs and minimum merge length of 400 bp. Filtering of low-quality sequences and dereplication to find unique sequences was done following default settings. OTU clustering and chimera removal was performed at a 97% identity threshold via the *cluster_otu* command implemented in the UPARSE algorithm. OTU sequences were taxonomically assigned by BlastN against the Silva SSU 138 database [43]. For the fungi reads, overlapping regions within paired-end reads were aligned to generate “contigs” by PANDAseq using default settings [44]. Taxonomic affiliations were assigned by BlastN against the UNITE database version 8.3 [45] with the Expect Value of 0.001, which was performed in a Galaxy workflow [46]. Dereplication, singleton removal and clustering of sequences to operational taxonomic units (OTU, >99% similarity) were performed using BLAST Parser [47] implemented in a Galaxy workflow.

### 2.5. Statistical Analyses

The multivariate analyses on the nematode and the nematode-associated microbial OTU abundances were carried out with the R software version R4.0.0 (R Core Development Team) using the packages vegan [48], labDSV [49] and in SAS 9.4 (SAS Institute Inc., Cary, NC, USA) using the GLIMMIX procedure [50]. The effect of replanting on the shoot or root fresh masses measured for each sampling was analyzed by one-way analysis of variance (ANOVA) with Tukey’s honestly significant difference (HSD) test. All statistical analyses were done on non-rarefied OTU data, but normalization of sequencing depth was based on relative abundances (i.e., sequence counts in each column were scaled by the column’s sum). The relative abundances of the nematodes were calculated on the order level and the associated microbes at the phylum level. The effect of soil type or treatment on the relative abundances was evaluated using a multivariate analysis of variance (MANOVA) followed by two-way ANOVA with Tukey’s HSD test. Diagnostic plots of residuals versus fitted values revealed the lack of significant heterogeneity of variance and Q-Q plots showed that assumptions of normality were justified. To test the effect of the treatment and the soil type on the nematode or the nematode-associated microbial communities, we fitted a Principal Component (PC) rotation test model using a SAS/IML script [51]. The squared multiple correlation coefficient R^2^ as effect measure can be interpreted as the proportion of variability in the observed similarity measures explained by the factor tested. LEfSe (Linear discriminant analysis Effect Size) was used to find differentially abundant OTU that best explain the differences between ARD and control plots in the community structures of nematodes and nematode-associated fungi and bacteria, respectively. The test first utilizes the non-parametric factorial Kruskal-Wallis (KW) sum-rank test [52] to identify OTU with significant differential abundance; biological consistency is afterward examined using Wilcoxon rank-sum tests [53]. Finally, LEfSe uses linear discriminant analysis (LDA) to estimate the effect size of each differentially abundant OTU (LSD > 2.0) [54]. To investigate the relationship between the differentially abundant nematode and nematode-associated microbial OTU and the root weight of apple plants, we used canonical correspondence analysis (CCA). Root weight was only available for each plant of samplings H4 (Heidgraben, April 2017) and E3 (Ellerhoop, April 2018). CCA was carried out on the relative abundances of OTU at 999 permutations. The correlation of OTU to the reduced plant growth was calculated by the “Envfit” function at 999 permutations [48].

## 3. Results

### 3.1. Reduction in Apple Plant Growth after Repeated Replanting

**Figure 1:**
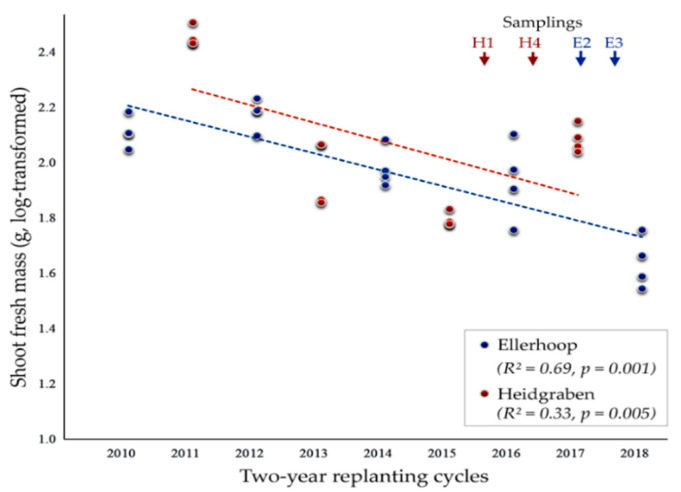
Reduction in shoot fresh mass of apple plants in the course of consecutive two-year replanting cycles in the experimental fields near Ellerhoop and Heidgraben. Shoot mass was determined after uprooting at the end of each growth cycle.

Replanting apple in the same plots resulted in progressive growth reduction in both fields, Ellerhoop and Heidgraben (Figure 1). In Ellerhoop, plant growth was generally poor in the first period ending in the year 2010, probably due to unfavorable weather conditions. However, shoot fresh mass was significantly reduced when considering the following periods of replanting. An exponential decline in shoot weight was observed. In Heidgraben, plant growth was drastically decreased in the first replanting period 2011–2013 and decreased further in the second period 2013–2015.

Despite on average good growth conditions in the period 2015–2017, which was also obvious for the plants in the Ellerhoop field, shoot mass was significantly lower compared to the first period. Overall, the growth decline associated with the replanting of apple rootstocks was faster and more severe in the more sandy soil of the Heidgraben field compared to the Ellerhoop field.

Root weight was measured for each plant of samplings H4 (Heidgraben, April 2017) and E3 (Ellerhoop, April 2018). Overall, the root weight was significantly reduced in replanted plots compared to plots that were first time planted with apple rootstocks (ANOVA, F = 21.9, *p* < 0.001). The effect was mostly attributed to sampling E3 with a 72% reduction of root weight in ARD soil, while plants sampled at H4 from ARD plots were only reduced by 30% in root weight compared to plants grown in control plots (Supplementary Figure S1).

### 3.2. Nematode Community Structure in Replanted and Control Plots

**Figure 2:**
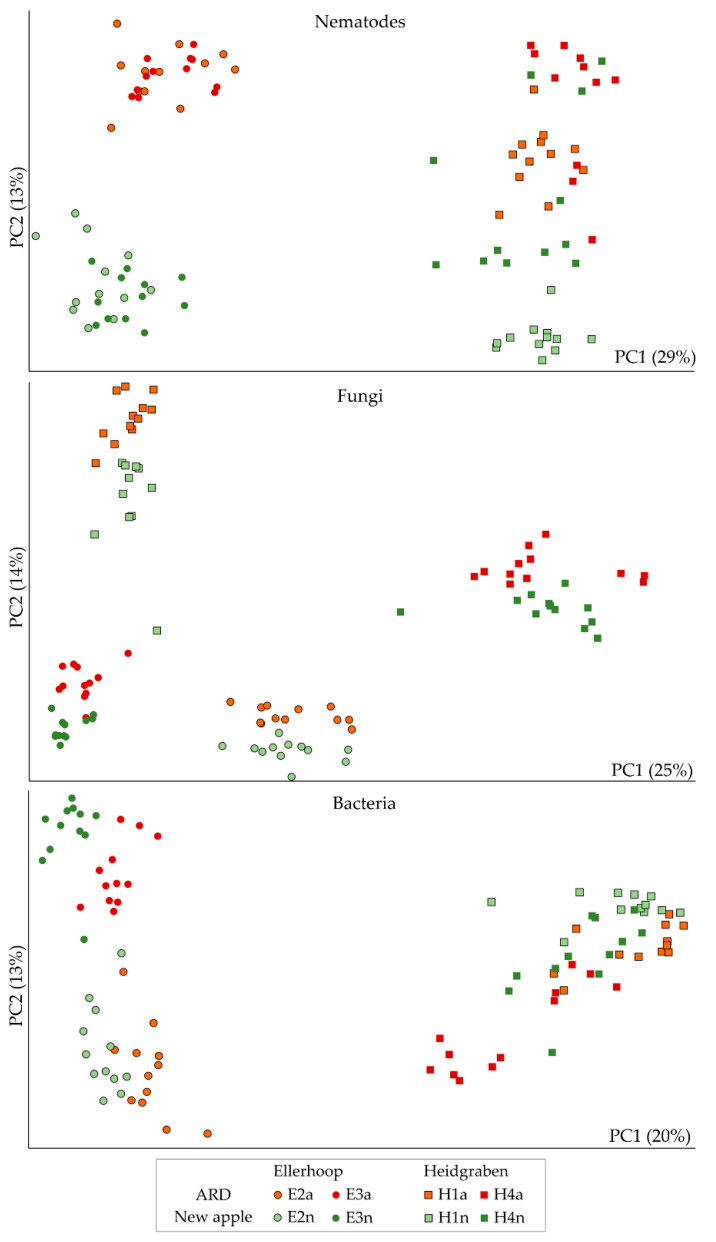
Principal component analyses of nematode communities and co-extracted fungal and bacterial microbiomes from replanted plots with apple replant disease (ARD, red/orange symbols) or control plots that were planted with apple for the first time after grass cover (new apple, green symbols). The field experiment of Ellerhoop was sampled in November 2017 (E2) and April 2018 (E3), the field experiment of Heidgraben was sampled in April 2016 (H1) and April 2017 (H4). The first two principal components (PC1, PC2, % explained variance in brackets) of log-transformed relative abundances of OTU from high-throughput amplicon sequencing data of ribosomal markers are shown.

Overall, high proportions of the orders Rhabditida and Tylenchida dominated the nematode communities, next to Triplonchida, Dorylaimida, Diplogasterida, Araeolaimida, Mononchida and Chromadorida (Supplementary Figure S2). The factors sampling and replanting significantly affected the relative abundance of most of these orders (Supplementary Table S1). On the level of OTU, replanting had a significant effect on the composition of the nematode community, when comparing replanted to control plots using a multivariate principal component test (Table 1). Nematode communities also differed significantly among samplings, reflecting the combined effects of soil type, site and year. However, replanting explained a greater proportion of the variance than the factor sampling, as indicated by a larger Pearson factor (Table 1). In principal component analysis, the second principal component clearly separated the nematode communities from replanted and control plots (Figure 2). While for the Ellerhoop field the nematode communities remained stable at consecutive samplings in plots of the same treatment, the nematode community structure in the Heidgraben field shifted in the direction of the second principal component. The first principal component separated the nematode communities from the two field sites.

Effect sizes of LDA determined by LefSe analysis revealed three nematode OTU that were strongly associated with replanted plots (Table 2). These were taxonomically assigned to the free-living genera *Cephalenchus* and *Aphelenchus* and a member of the Dorilaimida with an 18S rRNA sequence that has not yet been assigned to a known species. The bacterivorous nematode *Rhabditis* was strongly associated with the healthy control plots. The plant-ectoparasite *Helicothylenchus* was also associated with control plots, but less pronounced.

### 3.3. Nematode-Associated Fungi in Replanted and Control Plots

The taxonomic compositions of the fungal communities that were associated with the extracted nematodes were dominated by high proportions of Ascomycota and Basidiomycota (Supplementary Figure S3). Mortierellomycota were overall more abundant for each sampling in the nematode fractions that were extracted from replanted plots compared to control plots, although their abundance largely varied among samples. Rozellomycota and Chytridiomycota were significantly associated with replanted plots (Supplementary Table S1). The multivariate principal component test revealed, that replanting had a significant effect on the relative abundances of nematode-associated fungal OTU (Table 1). The composition of fungal OTU differed significantly among samplings (Table 1). This effect was more pronounced than the effect of replanting as indicated by a higher Pearson factor. In principal component analysis, the second principal component separated the nematode-associated fungal communities from replanted and control plots, while this separation was not as sharp as observed for the nematode communities (Figure 2).

Effect sizes of LDA determined by LefSe analysis identified *Mortierella* as significantly associated with the nematodes in replanted plots (Table 3). Less pronounced were associations with *Cercophora,* an unknown member of the Helotiales, and a species related to *Pseudogymnoascus*. Several OTU with similarity to species hypotheses of the UNITE database were indicative of control plots, mainly an unknown member of the family Chaetomiaceae with 100% sequence identity to species hypothesis SH1578257. With lower LDA scores, some nematode-associated fungal OTU related to the genera *Cirrenalia*, *Bipolaris*, *Marasmius* and *Metarhizium* were also indicators of healthy control plots.

### 3.4. Nematode-Associated Bacteria in Replanted and Control Plots

The phylum composition of the bacterial communities associated with nematodes varied by the sampling and to a lesser extent by the treatment (Supplementary Table S1). High proportions of Proteobacteria, Bacteroidetes and Actinobacteria dominated the nematode-associated bacterial communities. The composition of bacterial OTU that were co-extracted with the nematodes was significantly different between replanted and control plots (Table 1). Sampling (and thereby soil type/site/year) had a significant effect on the nematode-associated bacterial communities, which was more pronounced than the effect of replanting as indicated by a higher Pearson factor. In principal component analysis, the first principal component separated the nematode-associated bacterial communities of the two field sites (Figure 2). In contrast to nematodes and fungi, the first and second principal component explained a lower percentage of the total variance, and the second principal component did not well reflect the difference in replanted and control plots.

Lefse analysis pointed to the genera *Methylotenera*, *Methylophilus* and *Flavitalea* as indicators of replanting, although with much smaller effect sizes than observed for nematode and fungi indicators (Table 4). OTU belonging to the genera *Streptomyces*, *Sphingomonas*, *Acidibacter* or *Luedemannella* were most indicative of healthy control plots.

### 3.5. CCA Analysis of the Associations of OTU Abundance and Root Weight

For the later samplings E3 and H4, root weight was determined, which presumably reflected the effect of ARD on plant growth and the heterogeneous distribution of the disease among plots (Supplementary Figure S1). This gave the chance to examine the species vs. plant response relationship by CCA for these samplings. The analysis revealed that the nematodes with sequence similarity to *Cephalenchus hexalineatus* (R^2^ = 0.42, *p* < 0.001) and *Aphelenchus avenae* (R^2^ = 0.22, *p* < 0.001) and an unidentified Dorylaimida (R^2^ = 0.29, *p* < 0.001) were significantly associated with replanted plots at these two samplings. The co-extracted fungal genera *Mortierella* (R^2^ = 0.30, *p* < 0.001) and *Pseudogymnoascus* (R^2^ = 0.30, *p* = 0.038), and the nematode co-extracted bacteria *Methylophilus* (R^2^ = 0.23, *p* = 0.002), *Methylotenera* (R^2^ = 0.24, *p* < 0.001) and *Flavitalea antarctica* (R^2^ = 0.25, *p* < 0.001) were significantly more abundant in ARD soils and correlated significantly to root growth reduction. Thus, the results of CCA corresponded well with the results of the Lefse analyses over all samplings.

## 4. Discussion

Apple plants respond negatively to soils that have been repeatedly planted with apples. The yield decline is attributed to a yet unknown complex of soil biota [9]. Interactions of biological agents were proposed to cause ARD but the understanding of the interactions and their implication to the apple plant is deficient. Extracellular compounds released by fungi in the apple rhizosphere were shown to affect apple roots regardless of fungal colonization [15,19]. This gives evidence that biotic interactions in the rhizosphere of apple plants may lead to the external production of deleterious compounds, which trigger ARD but needs to be investigated thoroughly. Fungicide application to ARD soils resulted in improved plant growth and reduction of plant pathogenic fungi or oomycete complexes of *Cylindrocarpon*/Nectriaceae, *Fusarium*, *Pythium, Phytophthora* or *Rhizoctonia* [6,55,56,57]. However, their presence and frequency largely varied among orchards [58,59,60], and the contribution of some of the species such as *Pythium, Phytophthora* and *Rhizoctonia* were not confirmed as causal agents to ARD [61]. These previous studies presumed that ARD causal agents must invade the root, thereby excluding external chemical interference with biota in the rhizosphere. In contrast, the present study aimed to find nematodes and the nematode-associated microbes living freely in the plant-associated soil, which interact in a way that harms the roots of apple plants leading to the production of stress-induced compounds in the affected roots.

Here, the decline in shoot fresh mass measured over replant generations was used as a marker for ARD induction in the reference field sites. Generally, severe growth reduction was observed in Ellerhoop and Heidgraben thus indicating that ARD was induced at the sites. The progress of the disease was however different for each site. At Ellerhoop, plant growth was generally poor in the first period ending in the year 2010, probably due to weather conditions such as periods of drought, strong frosts or waterlogging, factors known to affect apple plant growth [32,62]. It however decreased drastically with each cycle of replanting, which is the peculiar characteristic of replant disease. In the sandy soil of the Heidgraben field, plant growth was drastically decreased in the first replanting period and decreased faster than in the loamy sand of the Ellerhoop field, which is in line with the observation of more severe ARD symptoms of apple plants in sandy soils compared to loamy soils [32].

In this study, the nematode composition at higher taxonomic order has been shown to vary among different soils or samplings rather than among the treatment. This observation reflects previous studies on the seasonal distribution of nematodes in apple orchards [63,64]. The order Rhabditida, constituting free-living nematodes was in high abundance compared to Tylenchida, which consists of mainly plant-parasitic nematodes. These findings support our hypothesis and coincide with our previous studies where it was shown that the free-living nematodes were significantly more abundant in ARD soils than the plant-parasitic nematodes [30]. Besides, other studies observed high proportions of free-living nematodes that varied amongst orchards [65]. The order Tylenchida constitutes major plant-parasitic nematodes that contribute significantly to global crop losses [66]. The groups’ role in the disease complex is subjective as their distribution in affected orchards is highly variable [8,67]. *P. penetrans* did not play a role in ARD of the investigated fields because it was low in abundance, it did not increase over time and it did not correlate with reduced root weight observed in the ARD plots. This finding is supported by studies by Manici et al. [17], who could not recover any plant-parasitic nematode from the roots of symptomatic apple plants. While Manici et al. [17] previously reported the presence of root-lesion nematodes in affected roots, the low frequency of these nematodes did not indicate any significant contribution to the growth reduction in apple trees.

Differences in nematode communities between ARD field plots and uncultivated grass plots were reported previously [30]. In this study, differences in species composition between ARD and healthy soils were shown irrespective of soil type or sampling time, in a way that confirms that the nematode communities associated with plants showing ARD symptoms may be truly unique and could be linked to the growth reduction observed in these soils. This was consistent when individual soils were compared as well and confirmed the minor effect of soil properties in the induction of ARD [33].

In E3 and H4 soils, plant growth was significantly higher in healthy than in replanted plots. Histological analysis carried out by one of the project partners on the fine roots of plants grown in the ARD soils showed noticeable indicators of the disease symptoms such as cell damages like necrosis, blackening and black cell inclusions in contrast to healthy roots [5]. We searched for putative OTU of nematodes and nematode-associated fungi or bacteria that were differentially abundant in the ARD across all sampling regimes (E2, E3, H1 and H4). We found OTU closely related to the nematodes *Aphelenchus avenae* and *Cephalenchus hexalineatus* and an unidentified Dorylaimida that were more abundant in ARD soils and correlated significantly with root growth reduction measured for soils E3 and H4, respectively. It must be noted that the short fragment from high-throughput amplicon sequencing does typically not resolve species, and databases are biased towards sequences of plant pathogens. *Aphelenchus* is a fungivorous nematode, which can potentially control plant-pathogenic fungi and in rare cases reproduce on seedling roots, callus tissue and moss [68]. *Cephalenchus* is an ectoparasite frequently found in tree nurseries or mostly associated with woody plants and was reported to feed on root cells [69,70]. *C. hexalineatus* was found to be pathogenic on roses [71] that belong like apple to the Rosaceae being susceptible to replant disease. Dorylaimida are commonly found in moist soils, especially around plant roots. Many of them are free-living nematodes feeding on bacteria and other microorganisms including soil fungi and algae [72]. Previous studies frequently found high abundances of bacterivorous nematodes, notably unidentified members of Rhabditidae, and *Cephalenchus* to be associated with peach or apple replanted soils [30,73].

Characterization of the microbial communities associated with the cuticle of nematodes has gained popularity over the years [74]. However, most studies focused on the microbes directly attached to the plant-parasitic nematodes in soils to understand the underlying mechanism of how microbes can antagonize these plant parasites [74,75]. There have been few studies of host-associated microbes on free-living nematodes but mostly focused on evolutionary and ecological studies [76,77,78]. In this study, the microbial fractions associated with the total nematode community have been characterized. OTU closely related to fungi of the genera *Mortierella*, *Cercophora, Pseudogymnaoascus* and unidentified Helotiales were associated with nematodes in ARD soils and correlated with reduced root weight. Notably, the genus *Mortierella* has been repeatedly linked with ARD soils from different orchards [79,80]. Species of *Mortierella* were reported to colonize apple roots, leading to the conclusion that they may be associated with the onset of ARD [55,81]. Members of the genus *Mortierella* produce metabolites with antifungal and antibacterial activity that may also affect root cells [82,83]. Recently, the genus was found to correlate negatively with shoot growth hence providing more evidence for their involvement in ARD [84]. In other cases, they are noted to antagonize plant-parasitic nematodes [85,86,87,88]. Whiles this fits our findings, some studies reported variable abundances of *Mortierella* in the root zone soils of apple trees that could not be linked to tree health or correlated negatively with the severity of ARD [61,79]. Some species of *Pseudogymnoascus* have been reported to cause death in animals and have been shown to produce cuticle-degrading subtilisin peptidases [89], which are used by nematode-trapping fungi to kill nematodes [90]. Species of *Cercophora* are reportedly found on dung and often produce antifungal metabolites that inhibit the growth of other fungi [91]. Their role in ARD may be linked to the production of deleterious compounds directed towards nematodes or fungi, which may induce a stress response in apple roots [56]. The order Helotiales contains some of the plant pathogens that cause brown rot of stone fruits, lettuce drop, black spot of roses, and soft rot of onions [92]. They were recently detected as the most dominant endophytes in ARD-affected roots [56]. Their role in ARD needs to be investigated further.

Bacteria OTU assigned to the genera *Methylotenera, Methylophilus,* or *Flavitalea* were more abundant in ARD soils and correlated to root growth reduction. However, the association of bacterial OTU with ARD was much less pronounced than nematode and fungal OTU, with respect to LDA effect size. *Methylotenera, Methylophilus* are both methanol consumers and frequently play a role in the denitrification process [93]. The genus *Flavitalea* was associated with the replant disease by negatively correlating with shoot growth in replanted soils [26].

In conclusion, our study showed that ARD soils differ in species composition compared to healthy soils. While cause-effect relation with symptoms of ARD cannot be proven, our results indicate that specific nematodes living freely in soil with their body-associated fungi or bacteria synergistically interact to affect apple plant health. Efforts will be made in the future to test putative nematode-associated microbe complexes in biotests for the pathogenicity of apple plants. In addition, extracellular compounds produced as a result of nematode–microbe interactions will be examined. Exploring the underlying synergy between the nematodes and microbes will give further insights into the etiology of the disease.

## Figures and Tables

**Table 1:** Multivariate tests on the effects of apple replanting on the nematode communities or nematode-associated microbes, with sampling (year/site) as confounding factor, using a number of principal components explaining 70% of the total variance.

Taxonomic Group	Fixed Effect	Pearson Factor	*p*-Value
Nematode community	Replanting (ARD/Healthy)	0.75	<0.0001
	Sampling (H1/E2/E3/H4)	0.56	<0.0001
Nematode-associated fungi	Replanting (ARD/Healthy)	0.56	<0.0001
	Sampling (H1/E2/E3/H4)	0.74	<0.0001
Nematode-associated bacteria	Replanting (ARD/Healthy)	0.52	<0.0001
	Sampling (H1/E2/E3/H4)	0.55	<0.0001

**Table 2:** Linear discriminant analysis (LDA) scores of differentially abundant nematode operational taxonomic units (OTU) that showed the highest association with apple replant diseased plots (negative scores) or control plots (positive scores) among all detected OTU, and their 18S rRNA sequence similarity to nematodes in the SILVA database (version 138.1).

OTU	LDA Score	SILVA BlastN Hit	Identity
36	−4.5	AY284594 *Cephalenchus hexalineatus*	100%
25	−4.2	AJ875139 Dorylaimida	99.6%
2	−4.2	AB368918 *Aphelenchus avenae*	100%
43	5.1	AY284653 *Rhabditis terricola*	100%
169	3.7	KJ869398 *Helicotylenchus digitiformis*	100%

**Table 3:** Linear discriminant analysis (LDA) scores of differentially abundant nematode-associated fungal operational taxonomic units (OTU) that showed the highest association with replanted plots (negative scores) or control plots (positive scores) among all detected OTU, and their ITS sequence similarity to nematodes in the UNITE database (version 8.3).

OTU	LDA Score	UNITE BlastN Hit	Identity
3184	−4.24	SH1557087 *Mortierella*	100%
4072	−3.65	SH1578257 *Cercophora*	100%
3099	−3.23	SH2750281 Helotiales	89.9%
4020	−3.22	SH1557243 *Pseudogymnoascus*	97.5%
3218	4.73	SH1615738 Chaetomiaceae	100%
1653	3.70	SH2732359 *Cirrenalia*	100%
4491	3.59	SH1657881 *Bipolaris sorokiniana*	100%
1503	3.54	SH2720643 *Marasmius*	96.9%
804	3.36	SH1561418 *Metarhizium marquandii*	99.6%

**Table 4:** Linear discriminant analysis (LDA) scores of differentially abundant nematode-associated bacterial operational taxonomic units (OTU) that showed the highest association with replanted plots (negative scores) or control plots (positive scores) among all detected OTU, and their 16S rRNA gene sequence similarity to bacteria in the SILVA database (version 138.1).

OTU	LDA Score	SILVA BlastN Hit	Identity
269	−3.08	EU937916 *Methylotenera*	98.7%
268	−2.56	KC172347 *Methylophilus*	99.5%
324	−2.42	KX146487 *Flavitalea antarctica*	100%
9262	3.11	DQ125809 *Streptomyces*	98.8%
38	2.94	MW339074 *Sphingomonas*	100%
284	2.89	EF019722 *Acidibacter*	98.5%
228	2.68	JF120108 *Luedemannella*	100%
1035	2.52	GQ264163 *Steroidobacter*	98.2%
2254	2.51	JF176556 *Chloroflexi*	99.5%

## Data Availability

All sequence data related to this study are available in the NCBI SRA database (Accession No. PRJNA749392).

## Supplementary Materials

The following are available online at https://www.mdpi.com/article/10.3390/microorganisms10010157/s1, Figure S1: Root fresh mass affected by replanting; Figure S2: Taxonomic profiles of nematode communities across all samplings in replanted plots and control plots (ARD/Healthy). The average relative abundances are shown on the order level. Taxa with less than 0.5% relative abundance were grouped as rare; Figure S3: Taxonomic profiles of nematode-associated fungal communities across all samplings in replanted plots and control plots (ARD/Healthy). The average relative abundances are shown on the phylum level. Taxa with less than 0.5% relative abdance were grouped as rare; Figure S4: Taxonomic profiles of nematode-associated bacteria communities across all samplings in replanted plots and control plots (ARD/Healthy). The average relative abundances are shown on the phylum level. Taxa with less than 0.5% relative abundance were grouped as rare; Table S1: Replanting effects on soil biota at order level.
