# Supplementary material for: Nematode–Microbe Complexes in Soils Replanted with Apple
Source: Microorganisms. 2022 Jan 12;10(1):157. doi: 10.3390/microorganisms10010157 (PMC8780120; doi:10.3390/microorganisms10010157)

Supplement

Nematode-microbe complexes in soils replanted with apple

Xorla Kanfra[1], Andreas Wrede[2], Julia Moll[3], Holger Heuer[1*]

[1] Institute for Epidemiology and Pathogen Diagnostics, Julius Kühn Institute (JKI) - Federal Research Centre for Cultivated Plants, Braunschweig, Germany

[2] Department of Horticulture, Landwirtschaftskammer Schleswig-Holstein, Ellerhoop, Germany

[3] Department of Soil Ecology, Helmholtz Centre for Environmental Research - UFZ, Halle (Saale), Germany

***Correspondence:**
Dr. Holger Heuer
holger.heuer@julius-kuehn.de

1 Supplementary Table

Supplementary Table S1| The effect of the treatment (replanted vs. control) or sampling on the relative abundances of major nematode orders and phyla of the nematode-associated microbes. Statistical analysis was done using a two-way analysis of variance (ANOVA) for sampling and treatment.

**Community**	**Order**	**Factor sampling (E2, E3, H1, H4)**			**Factor replanting (ARD- vs. control plot)**		
		**DF**	**F-value**	***P*-value**	**DF**	**F-value**	***P*-value**
Nematodes	Araeolaimida	3	12.68	<0.001	1	5.81	0.018
	Chromadorida	3	28.18	<0.001	1	0.86	0.357
	Diplogasterida	3	16.94	<0.001	1	4.04	0.047
	Dorylaimida	3	3.41	0.021	1	4.13	0.045
	Enoplida	3	0.42	0.738	1	1.28	0.262
	Mermithida	3	1.24	0.301	1	7.89	0.006
	Monhysterida	3	1.87	0.141	1	0.00	0.980
	Mononchida	3	6.79	<0.001	1	9.89	0.002
	Rhabditida	3	20.12	<0.001	1	20.72	<0.001
	Trichocephalida	3	3.12	0.030	1	0.01	0.938
	Triplonchida	3	41.75	<0.001	1	0.15	0.696
	Tylenchida	3	22.73	<0.001	1	22.16	<0.001
Nematode-associated fungi	Ascomycota	3	26.51	<0.001	1	1.49	0.226
	Basidiomycota	3	29.90	<0.001	1	0.93	0.337
	Chytridiomycota	3	11.04	<0.001	1	6.98	0.010
	Glomeromycota	3	3.96	0.011	1	0.00	0.955
	Mortierellomycota	3	22.13	<0.001	1	2.85	0.095
	Rozellomycota	3	11.25	<0.001	1	8.80	0.004
	Unidentified	3	0.62	0.6048	1	0.44	0.507
Nematode-associated bacteria	Proteobacteria	3	28.12	<0.001	1	2.01	0.159
	Bacteroidetes	3	16.53	<0.001	1	2.16	0.145
	unclassified	3	7.92	<0.001	1	5.59	0.020
	Actinobacteria	3	39.54	<0.001	1	0.00	0.999
	Acidobacteria	3	24.92	<0.001	1	9.01	0.003
	Firmicutes	3	10.06	<0.001	1	11.75	<0.001
	Verrucomicrobia	3	10.35	<0.001	1	13.65	<0.001
	Saccharibacteria	3	9.96	<0.001	1	2.21	0.141
	Chloroflexi	3	11.80	0.013	1	0.85	0.358
	Nitrospirae	3	8.65	<0.001	1	0.05	3.941

Degrees of freedom is denoted as DF. $P < 0.05$ indicates significant differences.

2 Supplementary Figures

Supplementary Figure S1. Root fresh mass of apple plants in response to ARD or healthy soils in the two reference sites Ellerhoop (E) and Heidgraben (H) measured during the 3rd and 4th sampling respectively. The line inside the box represents the median and the dots represent the samples (n=12). Significant differences are revealed by Tukey's test campared within each soil separately.

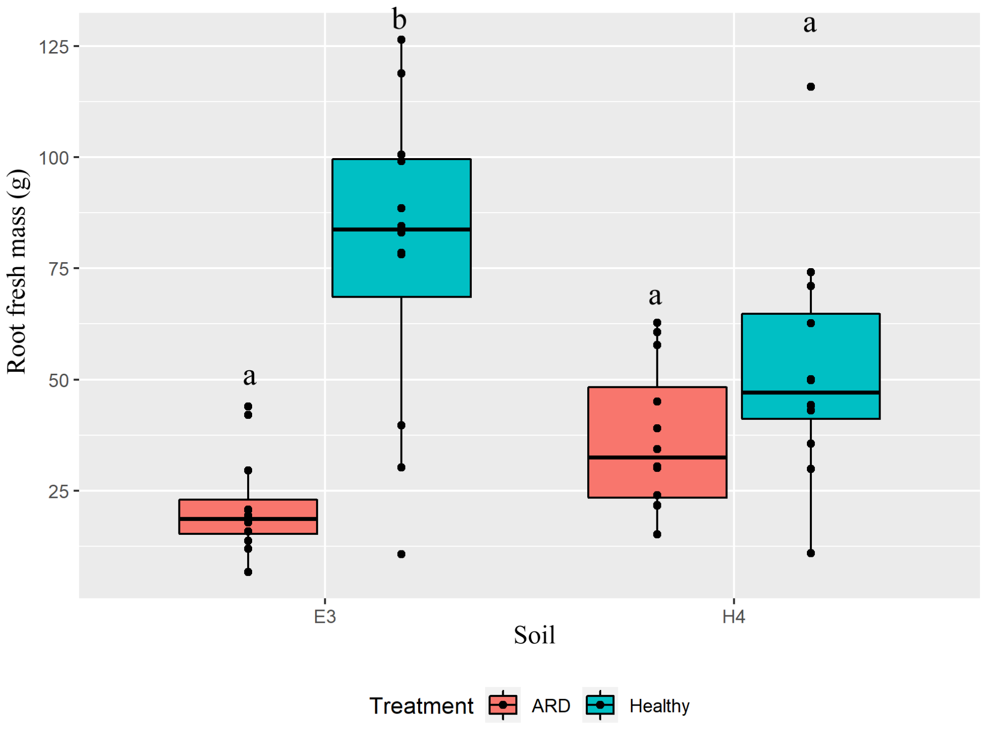

Supplementary Figure S2. Taxonomic profiles of nematode communities across all samplings in replanted plots and control plots (ARD / Healthy). The average relative abundances are shown on the order level. Taxa with less than 0.5% relative abundance were grouped as rare.

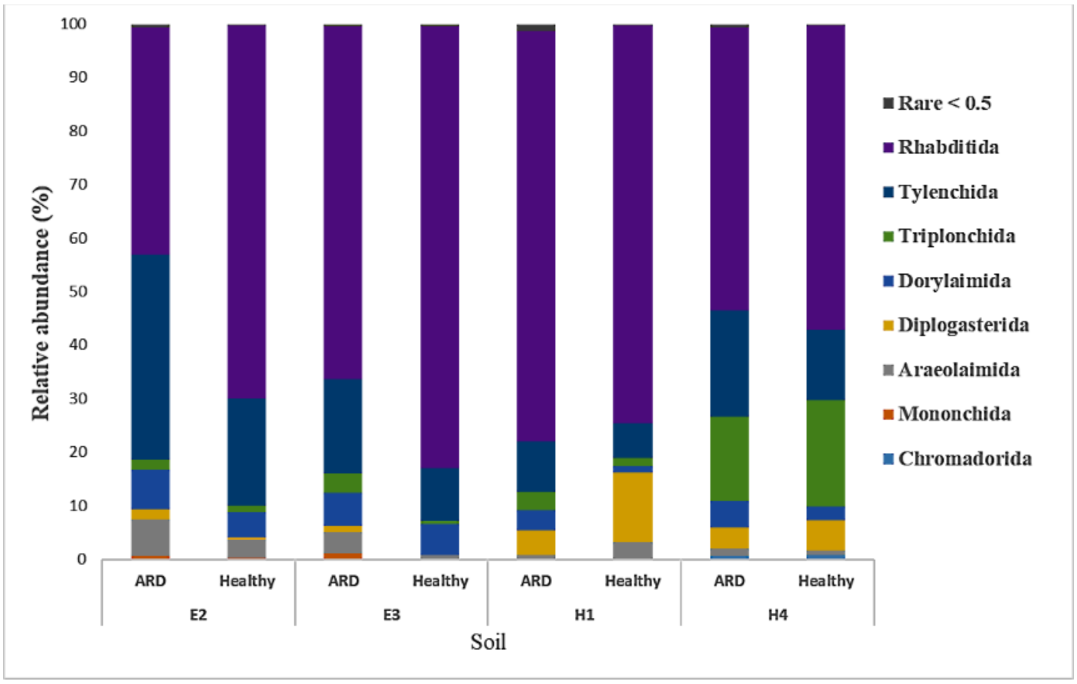

Supplementary Figure S3. Taxonomic profiles of nematode-associated fungal communities across all samplings in replanted plots and control plots (ARD / Healthy). The average relative abundances are shown on the phylum level. Taxa with less than 0.5% relative abundance were grouped as rare.

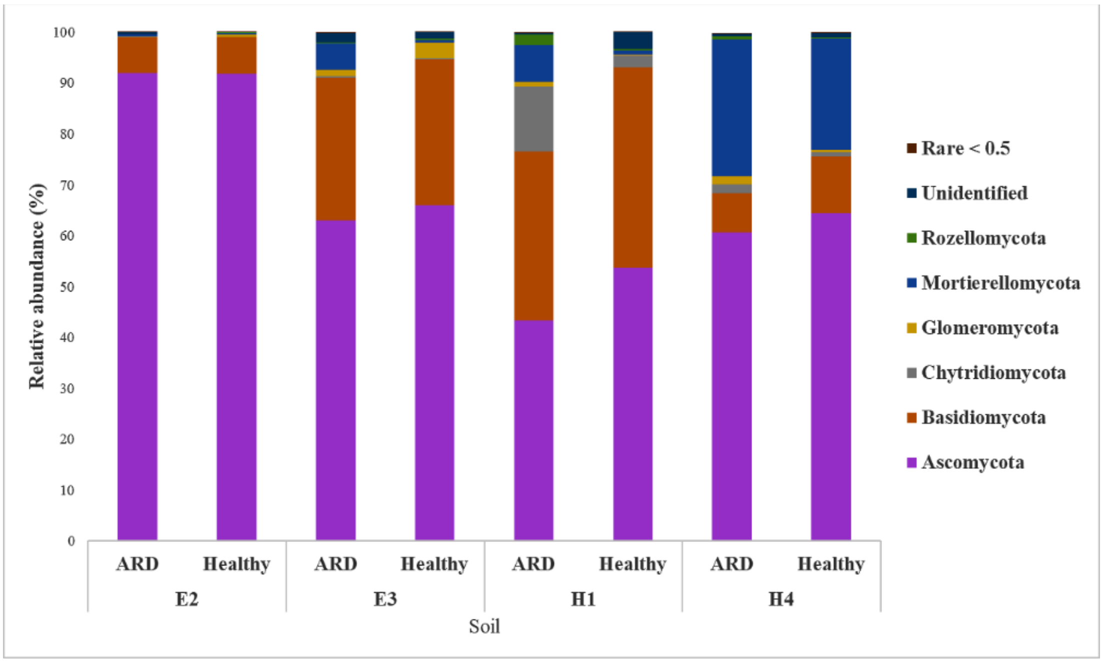

Supplementary Figure S4. Taxonomic profiles of nematode-associated bacteria communities across all samplings in replanted plots and control plots (ARD / Healthy). The average relative abundances are shown on the phylum level. Taxa with less than 0.5% relative abundance were grouped as rare.

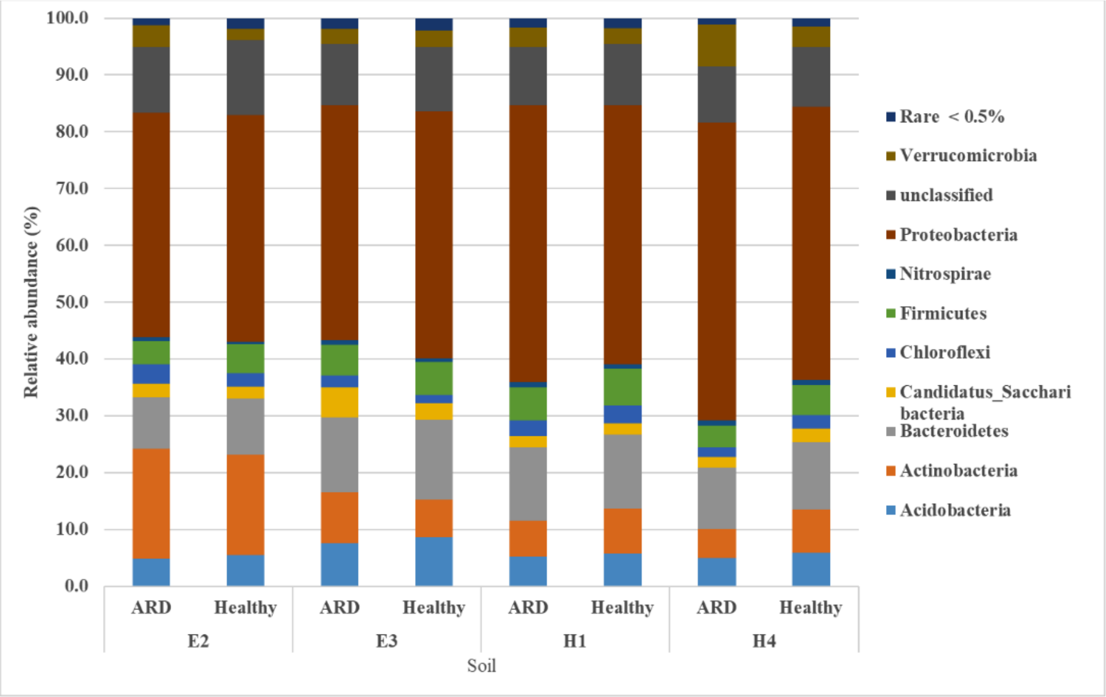

Supplement: Supplementary file 1 [file microorganisms-10-00157-s001.zip › microorganisms-1537253-supplementary.pdf]
